# Inactivation of Emerging Opportunistic Foodborne Pathogens *Cronobacter* spp. and *Arcobacter* spp. on Fresh Fruit and Vegetable Products: Effects of Emerging Chemical and Physical Methods in Model and Real Food Systems—A Review

**DOI:** 10.3390/foods14142463

**Published:** 2025-07-14

**Authors:** Junior Bernardo Molina-Hernandez, Beatrice Cellini, Fatemeh Shanbeh Zadeh, Lucia Vannini, Pietro Rocculi, Silvia Tappi

**Affiliations:** 1 Department of Agricultural and Food Sciences, University of Bologna, Piazza Goidanich 60, 47521 Cesena, Italy; beatrice.cellini2@unibo.it (B.C.); fatemeh.shanbehzade2@unibo.it (F.S.Z.); lucia.vannini2@unibo.it (L.V.); pietro.rocculi3@unibo.it (P.R.); silvia.tappi2@unibo.it (S.T.); 2 Interdepartmental Centre for Agri-Food Industrial Research, University of Bologna, Via Quinto Bucci 336, 47521 Cesena, Italy

**Keywords:** non-thermal processing, food safety, emerging pathogens, fruit and vegetables, essential oils, food safety, *Arcobacter*, *Cronobacter*

## Abstract

The consumption of fresh fruit and vegetables is essential for a healthy diet as they contain a diverse composition of vitamins, minerals, fibre, and bioactive compounds. However, cross-contamination during harvest and post-harvest poses a high risk of microbial contamination. Therefore, handling fruit and vegetables during processing and contact with wet equipment and utensil surfaces is an ideal environment for microbial contamination and foodborne illness. Nevertheless, less attention has been paid to some emerging pathogens that are now increasingly recognised as transmissible to humans through contaminated fruit and vegetables, such as *Arcobacter* and *Cronobacter* species in various products, which are the main risk in fruit and vegetables. *Cronobacter* and *Arcobacter* spp. are recognised food-safety hazards because they pose a risk of foodborne disease, especially in vulnerable groups such as newborns and immunocompromised individuals. *Cronobacter* spp. have been linked to severe infant conditions—notably meningitis and sepsis—most often traced to contaminated powdered infant formula. Although *Arcobacter* spp. have been less extensively studied, they have also been associated with foodborne disease, chiefly from dairy products and meat. With this in mind, this review provides an overview of the main chemical and physical sanitisation methods in terms of their ability to reduce the contamination of fresh fruit and vegetable products caused by two emerging pathogens: *Arcobacter* and *Cronobacter*. Emerging chemical (organic acid compounds, extracts, and essential oils) and physical methods (combination of UV-C with electrolysed water, ultrasound, and cold atmospheric plasma) offer innovative and environmentally friendly alternatives to traditional approaches. These methods often utilise natural materials, less toxic solvents, and novel techniques, resulting in more sustainable processes compared with traditional methods that may use harsh chemicals and environmentally harmful processes. This review provides the fruit and vegetable industry with a general overview of possible decontamination alternatives to develop optimal and efficient processes that ensure food safety.

## 1. Introduction

Foodborne diseases are responsible for significant morbidity in industrialised and developing countries, and fruit and vegetables are increasingly linked to foodborne disease outbreaks [1]. *Salmonella* and *Escherichia coli* strains are the most common etiological agents involved [2]. Some emerging pathogens, now increasingly recognised as transmissible to humans through contaminated fruit and vegetables, have received less attention such as *Arcobacter* and *Cronobacter* [3,4,5].

*Cronobacter* and *Arcobacter* spp. pose a new threat to fruit and vegetables as they can cause serious illness even at low levels of contamination. Products can be contaminated by *Arcobacter* spp. from the environmental, during processing, and by unhygienic handling. Due to its ability to form biofilms and resist disinfectants, they can persist in the processing environment. *Cronobacter* spp., particularly *C. sakazakii*, have been associated with fatal bloodstream infections, especially in infants and people with compromised immune systems.

The actual number of annually reported cases of infections caused by the *Enterobacteriaceae* family is not known. However, invasive *Cronobacter* infections in infants were added to the list of diseases in 2024. From January 2002 to July 2022, the Centres for Disease Control and Prevention (CDC) received 76 reports of severe *Cronobacter* disease in infants. About 40% of infants worldwide are infected with *Cronobacter* meningitis [3]. *Cronobacter* is a newly described bacterial genus that includes pathogens formerly known as *Enterobacter sakazakii. Cronobacter* spp. are capable of causing invasive diseases in all age groups but are mainly associated with meningitis, necrotising enterocolitis, and septicaemia in neonates [6]. Three *Cronobacter* spp. (*C. sakazakii*, *C. malonaticus*, and *C. turicensis*) have been identified as more virulent, capable of causing a high mortality rate (80%) in infants and are frequently isolated from cases of infant meningitis [3,4].

In 2022, 30 EU/EEA countries reported 140,241 confirmed cases of Campylobacteriosis. In 2018–2019, before the COVID-19 pandemic, reporting rates for Campylobacteriosis and *Cronobacter* infections were gradually declining. After a significant decline in case numbers in 2020, probably due to the pandemic, case numbers increased slightly in 2021 and remained stable in 2022 [7].

Bacteria of the genus *Arcobacter* are emerging as zoonotic pathogens transmitted through food [8]. *Arcobacter* spp. are Gram-negative (−) bacteria with a slightly curved shape, which may have a polar flagellum or bipolar flagella, and all species are able to grow under microaerobic conditions and are oxidase-positive. However, catalase is absent in some species [9]. This genus belongs to the family *Campylobacteraceae*, which includes two other genera: *Campylobacter* and *Sulfurospirillum*. *Arcobacter* species have been recovered from different environments and hosts, making this bacterial genus a large and diverse group of bacteria that currently includes 18 recognised species [10]. Some time ago, Vandamme et al. [11] proposed two species of the genus Arcobacter: *Arcobacter nitrofigilis* and *Arcobacter criaerophilus*. In 1992, the genus *Arcobacter* was expanded to include four new species: *A. butzleri* and a new species, *A. skirrowii*, which were isolated mainly from the preputial fluids of bulls or aborted foetuses of cattle, pigs, and sheep but also from diarrheal stools [12]. Since then, several new species have been described, mainly isolated from different environments and animals. Recently, six new species have been added to the genus, which now comprises eighteen species. *A. mytili* was the first species in the genus that could not hydrolyse indoxyacetate, which was isolated from mussels and brackish water in Spain [13]. *Arcobacter* is closely related to the genus *Campylobacter*, which belongs to the *Campylobacteriaceae* family. *Arcobacter* has an increased oxygen tolerance and the ability to grow at lower temperatures [12,13].

According to the CDC, the *Cronobacter* multispecies complex (formerly *Enterobacter sakazakii*) is a Gram (−) rod-shaped bacteria about 1 μm × 3 μm in size that belongs to the *Enterobacteriaceae* family. It has an oxidase-negative and catalase-positive phenotype and are facultatively anaerobic and motile due to peritrichous flagella [14].

The increased consumption of fruit and vegetable-based products is a growing concern, as plant foods are contaminated with microorganisms at various points during their processing, from the soil to the hands of the consumer.

Chemical sanitisers are used at various points in food processing because they are inexpensive and easy to use, prevent cross-contamination, have broad-spectrum antimicrobial activity, and a minimal negative impact on product quality [15,16,17,18]. However, the antimicrobial efficacy depends on the free chlorine content of the solution (50–200 mg/L chlorine), the pH value, and the organic/inorganic component [19]. Excess-free chlorine can cause a reaction with organic compounds and increase the concentrations of trihalomethanes and other toxic by-products [20]. Concerns about the formation of halogenated disinfection by-products when using chlorine for disinfection have focused attention on other disinfectants. It is therefore clear that highly toxic disinfection by-products can be formed. These by-products can harm the flora and fauna of the receiving bodies of water and negatively impair the microorganisms and plankton in these ecosystems. In environments where the water contains bromine (which occurs naturally as bromide), adding chlorine can lead to the formation of hypobromous acid/hypobromite through the oxidation of bromide [21].

Some studies have reported the efficacy of chemical sanitisers (chlorine, chlorine dioxide, and peroxyacetic acid as well as quaternary ammonium compounds (QAC)) on *Cronobacter* spp. [22] and *Arcobacter* spp. [23]. For example, Šilha et al. tested the survival of bacteria of the genus *Arcobacter* in several selected commonly used disinfectants and achieved complete inactivation. However, they also pointed out the potential risk of the secondary resistance of these pathogens to selected disinfectants [23]. On the other hand, Hausdorf et al. [24] reported the survival of *Arcobacter* spp. on the processing line of spinach even after repeated cleaning procedures.

Chlorinated water was effective in reducing *Cronobacter* spp. in fresh produce, but the survival depended on the specific product. According to Kim et al. [25], this pathogen is no more resistant than other enteric pathogens associated with fresh produce outbreaks.

Regarding the effects of thermal treatment, both *Cronobacter* spp. and *Arcobacter* spp. can be inactivated by thermal processes; however, consumer demand for improving the ‘fresh-like’ properties of processed foods such as juices and purees has prompted research into alternatives to thermal processing to avoid the detrimental impact of heat on the quality and nutritional value.

The impact of the food industry on the environment is of increasing importance to our society. Reducing the amount of pollutants generated by energy consumption, chemical sanitisers, and increasing the recycling of by-products are all requirements the industry must now fulfil. However, the development of new techniques in this area is being driven by ‘emerging technology’ concepts. These new technologies are able to replace traditional thermal and chemical treatments, which have a negative impact on the physical, nutritional, and bioactive properties of food, and are also more environmentally friendly.

Emerging technologies offer a powerful alternative. Like chlorine, new chemical alternatives effectively inactivate bacteria, viruses, and pathogens, replacing conventional chemical disinfectants, which are formulated with toxic substances that cause dangerous environmental impacts. Novel approaches based on chemical methods (organic acids, extracts, and essential oils) and non-thermal physical methods (ozone, pulsed electric field, catalytic intense pulsed light, cold plasma, electrolysed water, ultrasound, and food irradiation) [25,26,27,28,29,30,31,32,33,34,35,36,37,38,39,40] have been investigated for the decontamination of fruit and vegetable products.

However, while the effect of these technologies on the main spoilage and pathogenic species has been studied, there is little evidence on their effect on emerging pathogenic species. To date, there is no summarising overview of studies on the occurrence of *Cronobacter* and *Arcobacter* spp. in fruit and vegetables and the technologies used to inactivate them.

Although to our knowledge no outbreaks of infection with the two selected pathogens have been documented in association with fresh produce, it is believed that some outbreaks may remain undetected or poorly characterised, and the fact that there are multiple sources of transmission for these pathogens may make it difficult to unequivocally attribute infection to fruit and vegetable consumption [3]. Therefore, this review summarises the basic knowledge and current applications of emerging chemical and physical methods to inactivate the emerging pathogens *Cronobacter* and *Arcobacter* species, both of which are significant foodborne pathogens known for their resilience in food processing in fruit and vegetable products and model systems, thus contributing to an overview of the current methods and advances in the control of these microorganisms in food processing.

## 2. Incidence of *Arcobacter* spp. and *Cronobacter* spp. in Fruit and Vegetable Products

The high prevalence of *Arcobacter* and *Cronobacter* spp. in the agri-food industry can sometimes cause severe infection, diarrheal diseases, and occasional systemic infections such as bacteraemia and peritonitis in humans [41,42,43]. Due to its microaerobic nature, *Arcobacter* can colonise the intestinal tract of food-producing animals such as poultry, cattle, sheep, and swine [44]. However, the main route of infection has been identified as the consumption of contaminated food commodities such as unpasteurised dairy products, undercooked poultry meat, and/or contaminated water, vegetables, and fruits. However, limited studies exist on their prevalence in fruits and vegetables [42,43,44,45,46,47,48,49,50,51,52,53,54], as reported in Table 1.

Many studies are underway to find better methods for inactivating *Cronobacter* species, which are emerging foodborne pathogens [30]. Several foods, including fresh fruits, vegetables, cereals, milk, cheese, meat, and fish, have been shown to contain *Cronobacter* spp. [48,49,50,51]. *Cronobacter’s* primary ecological niche is probably plants [52]. This would account for the high predominance of salads (8.2%) and ready-to-eat vegetables (30.27%). *C. sakazakii* has often been isolated from fresh produce, and has shown the ability to survive and proliferate in fresh-cut fruits and vegetables stored in refrigerators at home or in retail environments at temperatures as low as 5.5 °C [25]. There is currently limited information regarding the prevalence of *Arcobacter* spp. in vegetables, particularly in ready-to-eat vegetables [42,43]. *Arcobacter*, for instance, was found in 27.5% of the vegetable samples that were ready to eat. Using biomolecular identification techniques, it was possible to identify 90.9% of the isolates as *A. butzleri* and 9.1% as *A. cryaerophilus* [46].

## 3. Emerging Strategies Tested for Decontamination of *Arcobacter* spp. and *Cronobacter* spp. in Fruit and Vegetable Products

**Figure 1:**
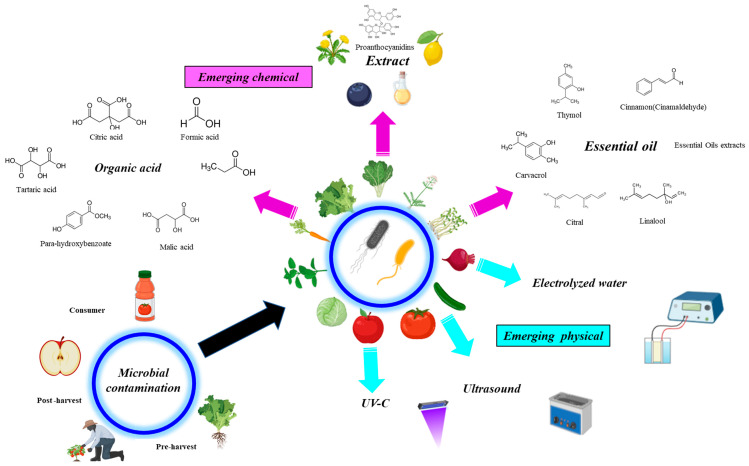
Emerging fruit and vegetable disinfection technologies to combat *Arcobacter* and *Cronobacter* species.

The alternative sanitising agents and new decontamination methods that have been tested against *Arcobacter* spp. and *Cronobacter* spp. are shown in Figure 1. These emerging technologies include chemical (organic acids, extracts, and essential oils) and physical methods (UV-C, ultrasound, electrolysed water) as well as their combinations for water disinfection at the lowest cost and minimal impact on fresh produce quality.

### 3.1. Emerging Chemical Methods

Emerging chemical methods (organic acid compounds, extracts, and essential oils) offer innovative and environmentally friendly alternatives to traditional approaches, which focus primarily on hazardous chemicals. These methods often utilise natural materials, less toxic solvents, and novel techniques, resulting in more sustainable and cost-effective processes. This section will discuss the literature findings regarding the use of organic acids, extracts, and essential oils as emerging chemical methods for decontaminating fresh produce from the selected pathogens.

#### 3.1.1. Organic Acid and Chlorine

Organic acids hold the generally regarded as safe (GRAS) status and are approved by the FDA and EC (European Commission). They are frequently used in the food industry as preservatives, antioxidants, flavourings, acidulants, and pH regulators [55]. Their mode of action is based on the acidification of the cytoplasm, dissociating and disrupting the cytoplasm’s pH and anion pool. This acidification can have an adverse effect on the cytoplasm and impair cell viability by compromising the integrity of purine bases and triggering the denaturation of important enzymes. Acidic ions can interfere with cell growth by increasing potassium ion transport into the cell. This results in glutamate export, cytoplasmic osmolarity disruption, and the denaturation of essential enzymes, negatively affecting cell viability [53]. Organic acids are effective in combating and reducing the incidence of pathogenic bacteria [53]. These include citric acid, formic acid, acetic acid, propionic acid, butyric acid, lactic acid, and fumaric acid [23]. The principal applications of organic acids and chlorine as emerging chemical methods for the inactivation of *Arcobacter* and *Cronobacter* spp. on fresh products are given in Table 2.

Červenka et al. [30] investigated the effects of pH and water activity (a_w_) on the growth of *Arcobacter butzleri* and *Arcobacter cryaerophilus* in a model system (i.e., BHI culture media at 30 °C). Different organic acids were used to achieve the target pH (formic, propionic, lactic, ascorbic, malic, tartaric, and citric acids) and different humectants were used to control a_w_. In general, propionic, lactic, malic, ascorbic (pH 5.5–5.0), formic, citric, and tartaric acids were more inhibitory to both *Arcobacter* species in the pH range of 6.0–5.5. Both *Arcobacter* strains were extremely sensitive to broths with a_w_ values of <0.980 using NaCl, glycerol, and sucrose as humectants. The authors conclude that sensitivity to a_w_ and pH could be an important prerequisite for the spread and survival of *Arcobacter* spp. in the environment, especially in food. In the same trend, Kim et al. [29] evaluated the effect of different acidic stress conditions (3.06, 4.00, and 5.02) and heat stress (55 °C) on *Cronobacter sakazakii*. According to the results, the acid-shocked *C. sakazakii* showed a higher resistance than the control group (pH 7.20) when the cells were exposed to an acidic pH. They found that *C. sakazakii* was acid-resistant but not alkali-resistant. The reason for this outcome could be that *C. sakazakii* is a Gram (−) bacterium with a high content of lipids in the cell wall, which react with an alkaline solution, leading to rupture of the cell membrane and cell death. In addition, *C. sakazakii* was more resistant to inorganic acids than to organic acids. The order of resistance of acid-shocked *C. sakazakii* to organic acids was acetic acid > propionic acid > malic acid. This adaptive response should be considered when deciding on control or disinfection methods for *C. sakazakii* in food or the environment.

On the other hand, some studies have shown that satisfactory results were observed when organic acids (peroxyacetic acid-based sanitiser-Tsunami 200) were tested in an in situ study. Kim et al. [25] applied them on the surface of fruit: apples, cantaloupes, strawberries, lettuce, and tomatoes stored at 4, 12, and 25 degrees °C for 8–28 days. The individual components chlorine and chlorine dioxide were able to reduce *C. sakazakii* on apples at a concentration of > or =50 ugml. The populations of *C. sakazakii* on apples treated with a sublethal concentration (10 microg/mL chlorine dioxide) for 1 or 5 min were reduced by 3.38 and 3.77 Log CFU/apple, respectively. Treatment with the Tsunami 200 disinfectant solution at a concentration of 40 microg/mL for 1 min resulted in a reduction of > or =4.00 Log CFU/apple. Treatment with 10 microg/mL chlorine or chlorine dioxide or 40 microg/mL Tsunami 200 for 5 min resulted in a reduction of > or =3.70 Log CFU/tomato. The reduction in populations of *C. sakazakii* on lettuce treated with 10, 50, and 100 micrograms/mL chlorine for 1 min ranged from 1.61 to 2.50 Log CFU/sample compared with the populations remaining on water-washed lettuce.

Gao et al. [31] evaluated the effects of para-hydroxybenzoate (BPB) on the thermal inactivation of *C. sakazakii* in commercial apple juice. They also assessed the effects of pH and possible synergistic effects with malic acid. BPB significantly enhanced thermal inactivation in a concentration-dependent manner, with D-values of a few seconds at the original pH (3.8). The BPB enhancement of thermal inactivation at pH 3.8 synergises heat resistance and malic acid, the primary organic acid of apple juice.

Some of the studies mentioned here show that the effect of organic acids varies depending on the type of fresh product, the target microorganism, the type of acid, and the treatment conditions. Despite the varying conditions and results, organic acids can be a promising emerging technology as they have beneficial results in food decontamination when it comes to reducing antimicrobials and pesticides. Synthetic fungicides can lead to the development of resistance in pathogenic bacteria, and chemical fungicide residues are a concern for human health.

#### 3.1.2. Extracts

Plants produce an invaluable source of secondary metabolites in response to environmental factors such as attack by herbivores, abiotic stress, or interspecific interactions [55]. Plant compounds such as carotenoids, polyphenols, terpenoids, and sulphur contain phytochemicals and alkaloids, which exhibit antibacterial properties [56]. Extracts can affect the cell membrane of Gram (+) and Gram (−) bacteria by inducing a decrease in cytoplasmic pH and hyperpolarisation of the cell membrane, indicating a possible mechanism of antibacterial action [57]. Examples of the application of plant-based extracts to fruit and vegetable products for decontamination from *Arcobacter* spp. and *Cronobacter* spp. are given in Table 3.

Chang et al. [39] investigated the antibacterial activity and mechanism of chrysanthemum bud crude extract (CBCE) against *C. sakazakii* and its application as a natural sanitiser. The antibacterial activity was evaluated by determining the diameter of the inhibition zone (DIZ), minimum inhibitory concentration (MIC), and minimum bactericide concentration (MBC). The CBCE extract showed DIZ, MIC, and MBC values against *C. sakazakii* of 14.55 ± 0.44–14.84 ± 0.38 mm, 10 mg/mL, and 20 mg/mL, respectively. In addition, the CBCE extract reduced approximately 6.5 Log CFU/mL of viable *C. sakazakii* in the biofilm on a stainless-steel tube, tinplate, glass, and polystyrene with 1 MIC of CBCE for 30 min at 25 °C. At the same time, the extract obtained from blueberry fruit showed an interesting antimicrobial activity that was due to the presence of strong antioxidant capacity associated with phenolic acids, catechins (flavonols), and proanthocyanidins (condensed tannins) [58]. Joshi et al. [33] evaluated the antimicrobial effects of blueberry proanthocyanidins (PAC) and commercial blueberry juice (BJ) against two strains of *C. sakazakii.* Reductions of approximately 1.50 Log CFU/mL were obtained after 30 min with BJ or blueberry PAC. Strains of *C. sakazakii* were reduced to undetectable levels from 8.25 ± 0.12 Log CFU/mL to 8.48 ± 0.03 Log CFU/mL, respectively, with BJ (pH 2.8) or blueberry PAC after 1 h. Moreover, the pH is a crucial parameter; when the cultures were treated with neutralised BJ for over 6 h, the suppression or inhibition of growth was not observed.

In the same trend, bioactive substances present in extra virgin oils, which are phenolic compounds, were investigated by Švarcová et al. [26] on two different extracts: unbuffered (WEOO) and buffered (BEOO). Extra virgin oil is an oil obtained using exclusively mechanical procedures, which grants the “virgin” with low free acidity (<0.8%), low peroxide value, high antioxidant activity, a perceptible fruity taste, and no sensory defects, acquiring in this way the label “extra”. Virgin olive oil is characterised as being obtained by mechanical processes (only washing, decantation, centrifugation, and filtration) under specific thermal conditions that do not cause any alteration. The in vitro results showed that the extra virgin olive oil extracts had the strongest antimicrobial effects, especially the unbuffered extracts, which showed strain inhibition after only 5 min of exposure. The weakest inhibitory effect was observed with olive oil extracts. In the environment of higher WEOO concentrations, a decrease in biofilm formation was observed, although at lower concentrations of the extracts, increased biofilm formation occurred due to stress conditions. Citrus oils, complex mixtures of natural compounds (approximately 400 compounds), have demonstrated antimicrobial activity against major foodborne pathogens [27,59,60,61,62,63,64,65,66]. Nannapaneni et al. [28] investigated the antimicrobial activity of seven orange oil fractions for their ability to inhibit the growth of selected *Arcobacter* spp. strains using the standard agar disc diffusion test. Cold-pressed terpeneless Valencia orange oil was the only fraction of six of the seven orange fractions with stronger inhibitory activity against Arcobacter strains (inhibition zones varying from 9.5 ± 0.7 to 29 ± 1.4 mm). To explain the antimicrobial activity, the authors used gas chromatography-mass spectrometry (GC-MS) to identify the presence of linalool (20.2%), which appears as the dominant component of this fraction, followed by decanal (18%) and geranial (9.1%), which induce the inhibitory effect of these molecules through their interaction with the structural components of the bacterial cells.

**Table 3:** Application of extracts and essential oils as emerging chemical methods for the inactivation of *Arcobacter* and *Cronobacter* spp.

Microorganism	Extract and Essential Oil	Matrix	Condition	Microbial Reduction	Potential Research Limitation	Reference
*Arcobacter* spp.	Extract virgin olive oils	In vitro test	Non-buffered (WEOO) and buffered (BEOO) extract virgin olive oils.	Reduction of 5 Log CFU/mL.	The effect of extract virgin olive oils on biofilm eradication has not been evaluated.	[26]
*Arcobacter* spp.	Orange oil fractions	In vitro test	Seven commercial orange oil fractions.	No inhibition of *Arcobacter* spp. was detected by 6 out of 7 orange fractions except CP terpeneless Valencia orange oil, which produced inhibition zones varying from 9.5 ± 0.7 to 29 ± 1.4 mm.	Composition of the tested orange-based fractions was not determined.	[28]
*Cronobacter sakazakii*	Blueberry proanthocyanidin (PAC) and commercial blueberry juice (BJ)	In vitro test	Blueberry PAC at 5 mg/mL, BJ (pH 2.8), neutralised BJ (pH 7), malic acid (pH 3.0), or PBS and incubated at 37 °C for 30 min, 1 h, 3 h, or 6 h.	Reductions of ~1 and 1.50 Log CFU/mL with BJ or blueberry PAC. Reduction of 8.25 ± 0.12 Log CFU/mL and 8.48 ± 0.03 Log CFU/mL, respectively, with BJ (pH 2.8) or blueberry PAC after 1 h, while malic acid (pH 3.0) showed 1.3 Log CFU/mL reduction for both strains.	Composition of blueberry polyphenolic fractions in commercial blueberry juice and blueberry proanthocyanidin (PAC) fraction has not been characterised.	[33]
*Cronobacter sakazakii*	Chrysanthemum bud crude extract (CBCE)	In vitro test	Different concentrations of CBCE (0, 0.3125, 0.625, 1.25, 2.5, 5, 10, and 20 mg/mL).	Diameter of inhibition zone (DIZ), minimum inhibitory concentration (MIC), and minimum bactericide concentration (MBC) of CBCE against *C. sakazakii* were 14.55 ± 0.44–14.84 ± 0.38 mm, 10 mg/mL, and 20 mg/mL, respectively.	The composition (principal bioactive compounds) of the chrysanthemum bud crude extract (CBCE) has not been determined.	[39]
*Cronobacter* spp.	Bacterial cellulose (BC) impregnated with plant extract and essential oil	In vitro test	Bacterial cellulose combined with extracts (Tulsi, Brahmi, lemon, blackberry, nettle root, and nettle leaf) and essential oils (cinnamon, sage, clove, mint, thyme, lemongrass, rosemary, lemon, anise, tea tree, lime, grapefruit, and tangerine) in agar diffusion methods.	The cellulose matrix with a 50% extract from Brahmi was found to effectively inhibit the growth of the selected *Cronobacter* strains.	Composition of the extract and essential oil fraction was not determined.	[63]
*Cronobacter* spp.	Biocellulose impregnated with oregano essential oil	In vitro test	Biocellulose impregned with oregano essential oil (100%) in agar diffusion methods.	Bacterial cellulose impregnated with oregano essential oil had strong and moderate antimicrobial activity against all presented strains of the genus *Cronobacter*.	Composition of the extract and essential oil fraction was not determined.	[64]
*Cronobacter* spp.	Thyme, cinnamon, clove, peppermint, marjoram, cumin, rosemary, fennel, basil, lime, bergamot orange, orange, lemon, grapefruit, mandarin, cardamom, anise, and ginger	In vitro test	Disc-diffusion method was used to screen the EOs.	Most effective EOs: thyme > cinnamon > marjoram. The clove, cumin, and fennel oils had moderately inhibiting effects on only some of the tested strains.	The effect of the essential oils on biofilm eradication was not evaluated.	[65]

#### 3.1.3. Essential Oils

Other natural antimicrobial agents from plant sources, such as essential oils (EOs), have been used for centuries to preserve food and improve flavour. Several plant-derived essential oils have been shown to have antimicrobial properties, and a variety of their active components have been identified [67]. The main mechanism of action of essential oils against microorganisms is the interaction of their hydrophobic components with the cell membrane lipids. This interaction leads to a loss of membrane integrity, and this damage causes changes in the function of the electron transport chain, nutrient uptake, protein and nucleic acid synthesis, the coagulation of cell contents, and the inhibition of enzymes important for energy metabolism, leading to cell death [68].

Examples of the application of essential oils to fruit and vegetable products for decontamination from *Arcobacter* spp. and *Cronobacter* spp. are reported in Table 3. Anna Berthold-Pluta et al. [65] evaluated the effect of EOs from thyme, cinnamon, clove, peppermint, marjoram, cumin, rosemary, fennel, basil, lime, bergamot, orange, lemon, grapefruit, mandarin, cardamom, anise, and ginger against 21 strains of *Cronobacter* species. The in vitro test using the disc diffusion method showed that the most effective EOs were thyme > cinnamon > marjoram. The EOs generally showed high antibacterial activity against Gram (+) bacteria and lower activity against Gram (−) bacteria. Such differences have been related to the limited migration of the hydrophobic substances into the cytoplasm due to the presence of lipopolysaccharides around the bacterial peptidoglycan layer. The author also determined the maximum tolerable concentration (MTC) and minimum inhibitory concentration (MIC) for the four active compounds of the essential oils: thymol, trans-cinnamaldehyde, eugenol, and menthol. The results showed that the strains were tolerant to thymol (between 0.013 and 0.025%) and exhibited strong sensitivity when treated with trans-cinnamaldehyde (between 0.025 and 0.1%). The tolerance of the tested strains to eugenol varied between 0.05 and 0.1%, and menthol proved to be the EO component with the weakest antibacterial activity. The authors attributed the strong antimicrobial effect of trans-cinnamaldehyde to the downregulation of F1F0-ATPase, which leads to the inhibition of ATP synthesis, disrupts the cell membrane, and alters the lipid profile of the membrane. Thymol may disrupt the cytoplasmic membrane of a cell, increasing its permeability and depolarising its potential. Eugenol and menthol can migrate through the aqueous extracellular medium and interact with and damage lipid membranes. On the contrary, Fisher et al. [27] reported using essential oils of lemon, orange, and bergamot against three strains of *A. butzleri*. In in vitro tests using the disc diffusion method, limonene essential oil or vapour did not affect any tested strains. Additionally, lemon and orange oils were less effective than bergamot, citral, and linalool in inhibiting bacterial growth. Because extracts sometimes contain various chemical components, it is challenging to attribute an essential oil’s antimicrobial activity to just one or a few of its active ingredients. In addition to the main ingredients, others present in smaller or perhaps even negligible concentrations may also have a key role in the antibacterial qualities of EOs.

Another alternative is the use of EO compounds in the production of active packaging with antimicrobial properties. For example, biocellulose is a natural and environmentally friendly polymer that selected acetic acid bacteria strains can produce. In a study, Nagmetova et al. [64] investigated whether biocellulose impregnated with natural oregano essential oil has antibacterial activity against *Cronobacter* spp. strains. Bacterial cellulose from the strain *Komagataeibacter* GH1 with added oregano EO generally showed moderate to vigorous antimicrobial activity against all of the tested strains of the genus *Cronobacter* spp. isolated from the plant matrix. In the same trends, Stasiak-Różańska et al. [63] investigated the antimicrobial effect of biocelluloses produced by Gluconacetobacter hansenii ATCC 23769. These were used as a matrix for extracts (Tulsi, Brahmi, lemon, blackberry, nettle root, and nettle leaves) and essential oils (cinnamon, sage, clove, mint, thyme, lemongrass, rosemary, lemon, aniseed, tea tree, lime, grapefruit, and tangerine). The authors found that the cellulose matrix containing 20% of the extract showed no growth inhibition zones. However, 50% of the solutions of these extracts were prepared and analysed in the next phase of the study. The authors found that the 50% extract of Brahmi effectively inhibited the growth of the selected *Cronobacter* spp. strains. The other extracts showed no antimicrobial activity against the tested strains.

### 3.2. Emerging Physical Methods

Among the emerging physical methods for the decontamination of fresh produce, this section will discuss the literature findings regarding the combination of UV-C with electrolysed water and ultrasound with organic acid treatments. Examples of the application of emerging methods to fruit and vegetable products for decontamination from *Arcobacter* spp. and *Cronobacter* spp. are given in Table 4.

#### 3.2.1. Combination of UV-C with Electrolysed Water

Several alternative techniques, including electrolysed water (EW) (neutral—NEW—or acidic—AEW) and short-wave ultraviolet-C (UV-C) illumination, have been used as preservation and decontamination methods [69]. Electrolysed water (EW) has received considerable attention in recent years as a new disinfectant [70]. The main reason for its popularity is the simplicity of its production and application and the fact that it has no adverse effects on the environment compared with other disinfectants. The disinfectant effect of EW depends on three factors: a low pH value, a high oxidation-reduction potential (ORP), and the synergistic effect of HClO, Cl_2_, H_2_O_2_, and hydroxyl (OH−). A low pH value impairs the permeability of cell membranes. A high ORP value impairs the metabolic compounds in the bacterial cells and leads to cell death. OH− and H_2_O_2_ can damage the lipid membranes of the cells, denature proteins, and prevent the cells from reproducing. This combination destroys the bacteria by altering the DNA, thus preventing enzyme activation [71,72].

Furthermore, Graça et al. [69] proposed substituting sodium hypochlorite solutions with UV-C to decrease microbial populations. According to the hurdle technology, combining sanitising treatments could have a synergistic (sodium hypochlorite) effect, leading to better microbial reduction. Alternatively, lengthening the shelf life of kiwifruit [70], apples [69], apricots [73], melon [74], mango and pineapple [75], pear [69], and watermelon [76] has been explored. Santos et al. [37] investigated the growth capacity of *Cronobacter sakazakii* and the efficacy of ultraviolet-C (UV-C) radiation, acid electrolysed (AEW), and neutral electrolysed (NEW) water in inhibiting these bacteria on minimally processed ‘Tommy Atkins’ mangoes by combining sanitising treatments. Fruits were contaminated by dip inoculation and stored for 10 days at 4, 8, 12, and 20 °C while the bacteria were counted. Contaminated mangoes were disinfected with different UV-C irradiation (2.5, 5, 7.5, and 10 kJ/m^2^). At 4 °C, 8 °C, and 12 °C, the enterobacteria population decreased in the first 48 h and remained almost unchanged until the end of the test, regardless of whether it had survived during the 10 days. At 20 °C, the population inoculated at 4.6 Log CFU/g decreased to 3.2 Log CFU/g on the first day and then grew exponentially to a population of 7.2 within two days. The results described in the present work show that fresh-cut ‘Tommy Atkins’ mangoes allowed for the growth of both Enterobacteriaceae at 20 °C but not at 4 °C, 8 °C, or 12 °C during the period studied. In general, UV-C was more effective in reducing *C. sakazakii* (2.4–2.6 Log CFU/g) compared with AEW, NEW, and SH (1.2–1.8 Log CFU/g). The authors agreed on the importance of considering the different survival mechanisms of microorganisms to protect themselves and the importance of the food matrix when investigating and testing different decontamination technologies. The greatest challenge with this technology is likely to be its compatibility within a continuous food processing process and the duration time of the UV irradiation of these foods without causing any change to the product.

#### 3.2.2. Ultrasound

Ultrasound has been identified as a potential physical technique with antimicrobial activity. Ultrasound treatment can cause a range of physical damage to the membrane of living cells to varying degrees, most notably membrane perforation and increased membrane permeability. This phenomenon of membrane perforation is mainly related to the mechanical effects caused by the collapse and oscillation of acoustic cavitation bubbles and the shear stress of the microcurrents around the bubbles [77].

Indeed, in 2016, Park et al. [34] investigated the synergistic effect of a combination of ultrasound (37 kHz, 380 W for 5–100 min) and sodium hypochlorite (NaOCl) (50–200 ppm) on the reduction in *Cronobacter sakazakii* contamination in lettuce. Ultrasound was not sufficient to inactivate *C. sakazakii* (0.58 log), while NaOCl significantly (*p* < 0.05) reduced *C. sakazakii* (2.77 Log_10_ reduction). Despite the significant reduction in *C. sakazakii* by NaOCl treatment (200 ppm), the combination of 100 min of ultrasound and 200 ppm NaOCl resulted in an additional 1.67 Log_10_ reduction in *C. sakazakii* (4.44 Log_10_ reduction). The micrographic SEM images showed that the combination of both treatments led to a change in the lettuce surface: a smooth surface, although some cells remained, and a drastic reduction in bacterial populations compared with a single treatment. Other chemical compounds used in conjunction with extended ultrasound include peroxyacetic acid, an alternative to reduce the use of chlorinated disinfection by-products such as trihalomethanes (THMs), which are potentially harmful to health in high concentrations, similar to carcinogens [78].

Bang et al. [35] tested the synergistic effect of a combination of ultrasound (37 kHz, 380 W for 10–60 min) and peroxyacetic acid (50–200 ppm) on the reduction in *Cronobacter sakazakii* biofilms on cucumbers. Ultrasound (US) was not sufficient to eliminate biofilms (0.60 Log_10_ reduction), while PAA (200 ppm) significantly (*p* < 0.05) reduced the biofilm formation in cucumbers (1.88 Log_10_ reduction). Furthermore, the combination of 60 min US and 200 ppm PAA resulted in an additional 3.51 Log_10_ reduction. The micrographic SEM images showed that, in this case, PPA in combination with US treatment reduced the biofilm populations compared with the SEM images obtained with the combination of NaOCl and ultrasound. *C. sakazakii* were loosely aggregated and scattered due to the cavitation of ultrasound, while they were destroyed in regular forms due to the antibacterial effect of PAA.

#### 3.2.3. Methods Not Explored: Cold Plasma as an Emerging Technology for Decontamination of Fruit and Vegetable Products

In the literature, only very few reports have addressed the decontamination of *Cronobacter* spp. in dry food and food contact materials by cold plasma, but none of *Arcobacter* and none specifically targeting the decontamination of fruits and vegetables. However, since this non-thermal technology shows great potential for decontaminating fresh produce, it was included in this review.

In particular, cold atmospheric plasma (CAP) is an emerging non-thermal process that offers numerous potential applications in food processing and environmental protection. In the food industry, the cold plasma process effectively treats fruits and vegetables by breaking down organic matter and reducing microbial pollutants, enhancing food safety, and extending shelf life [79]. CAP comprises a range of biologically active substances such as ozone, reactive oxygen species, free radicals, and nitrogen dioxide. These active substances undergo physical and chemical reactions with microbial cell membranes, resulting in the rupture of the cell membrane structure and cell death caused by plasma radicals [80,81].

The three significant challenges to the widespread adoption of atmospheric plasma technologies as a food-manufacturing tool are (1) biosecurity of the CAP treatment on foods, (2) designing the plasma source, and (3) process control. Therefore, further research on the genotoxic/cytotoxic effect of CAP treatment is needed to understand the chemical reaction and mechanism of plasma and its by-products in food. Therefore, the operating conditions of plasma treatment should be optimised to meet the safety standards, just like other processing technologies [82].

In recent years, some research has been conducted to investigate the potential of CAP to inactivate a variety of *Cronobacter sakazakii* strains, but only in in vitro and dry food products. For example, Phan et al. [83] demonstrated the inactivation of *C. sakazakii* biofilms on three food contact surfaces (stainless steel, silicone, and PVC) using high-voltage atmospheric cold plasma (HVACP) applied as a dielectric barrier discharge in a helium-modified atmosphere. After a 90-s exposure to cold plasma at 40 kV, *C. sakazakii* was reduced by ∼3 Log CFU/coupon compared with the treatment without cold plasma. Chen et al. [84] evaluated the efficacy of CAP on *C. sakazakii* in non-fat dry milk powder using a fluidised bed reaction system. The CAP treatments for 20–120 s reduced *C. sakazakii* by 1.17–3.27 Log_10_. The inactivation of *C. sakazakii* increased with an increasing flow rate of 8 to 20 L/min. In another study, *C. sakazakii* in onion powder and powdered infant formula was reduced to 0.6 and 0.9 Log CFU/g by cold plasma treatment at 900 W for 20 min [85]. However, no study has compared the inactivation rates of different food groups using the same cold plasma treatment system (e.g., fruit and vegetables).

These previous studies suggest that the method of plasma generation, the application, and the variability among species and strains have an impact on the efficacy of CAP against pathogenic bacteria. Actually, CAP is designed to inactivate other foodborne pathogens on the surfaces of fresh produce. For example, the effect of cold plasma on the inactivation of *Escherichia coli*, *Salmonella*, and *Listeria monocytogenes* artificially inoculated on apples, melons, and lettuce was evaluated [86,87]. Several authors have hypothesised that these Gram (+) organisms are more resistant to plasma treatment than Gram (−) organisms. This phenomenon, referred to as the etching effect, is due to the properties of the charged particles and the reactants involve highly reactive free radicals, such as reactive nitrogen and reactive oxygen species, which can undergo intense chemical interactions with the cell membrane. For this reason, it is important to conduct new research in the future aimed at investigating treatments with CAP against *Arcobacter* and *Cronobacter* species and their resistance as Gram (−) strains.

## 4. Conclusions and Future Perspective

The food industry has a responsibility to society, as it meets all of the world’s food needs, and needs to guarantee safe foods to all consumers. As mentioned in this overview, food safety is one of the most important priorities in processing fruit and vegetables. However, harvesting conditions (e.g., fertilisers and irrigation water) and post-harvest and processing operations (e.g., cross-contamination) can favour contamination with microorganisms resistant to traditionally used antimicrobials, which has been one of the major problems in recent years [88]. The results of the conventional and new strategies developed to date show different efficacy ranges against the two emerging pathogen species, which were discussed in this comprehensive review. However, these techniques have aspects that need to be improved for large-scale industrial applications.

In the specific case of the chemical and physical technologies described, their positive effect on disinfection is undeniable, but the exposure time and concentration are crucial. It is also necessary to determine whether the chosen disinfection strategy and the combinations between them require additional preservation measures to assure food safety and guarantee the extension of the valuable life of fruits and vegetables. It is important to understand that if the technology used does not achieve the desired level of disinfection, microorganisms can survive through defence mechanisms and affect the quality of the product. In this context, preventing the survival and spread of highly resistant strains and resistance to disinfectants has become a global strategic concern. Because there is insufficient research on the types of disinfectants and the target bacteria do not comprehensively represent the whole, it is challenging to elucidate the primary resistance mechanism of bacteria to disinfectants in various real-world situations.

On the other hand, the consumers’ acceptance of new food technologies is a complex issue, and the research suggests that the importance of particular attributes will vary based on the specific information provided to consumers. For example, how consumers view the advantages and disadvantages, cost, and perceived “naturalness” will influence their acceptance of genetic modification and food nanotechnology. Another aspect that must be considered is the shape of the products to be processed, mainly because there are technologies whose limits are related to the low penetration capacity of the treatment. Ongoing studies on this technology prove that it is strongly effective for surface decontamination, with efforts in progress for liquid processing. The limited low penetration of this technology is also a major hurdle to its applicability, as the microorganisms lurking beneath the effective penetration depth of the compounds may be spared. Finally, the operating parameters must be optimised to maintain product quality while complying with the food safety criteria for relevant foodborne bacteria. In the meantime, the investment and costs for large-scale applications should be analysed.

## Figures and Tables

**Table 1:** Reports on the occurrence of *Arcobacter* spp. and *Cronobacter* spp. in fruit and vegetable products.

Food Matrix	Genus	Occurrence	Reference
Lettuce	*Arcobacter* spp.	-	[4]
Alfalfa broccoli	*Cronobacter sakazakii*	21/60	[38]
Small radish	*Cronobacter muytjensii*		
Lentil	*Cronobacter turicensis*		
Sunflower	*Cronobacter malonaticus*		
Leek and sprout mix	*Cronobacter condiment*		
Rucola	*Cronobacter sakazakii*		
Lamb’s lettuce	*Cronobacter muytjensii*		
Endive escarole	*Cronobacter turicensis*		
Leaf vegetables mix	*Cronobacter malonaticus*		
Lettuce	*Arcobacter butzleri*	16/110	[41]
Rocket	*Arcobacter butzleri*		
	*Arcobacter cryaerophilus*		
Carrot	*Arcobacter skirrowii*		
	*Arcobacter butzleri*		
Beet root	*Arcobacter skirrowii*	28/204	[42]
	*Arcobacter butzleri*		
Cabbage	*Arcobacter butzleri*		
Lettuce	*Arcobacter* spp.	21/90	[44]
Spinach			
Arugula			
Lettuce	*Arcobacter butzleri*	44/160	[46]
Spinach	*Arcobacter cryaerophilus*		
Rocket	*Arcobacter butzleri*		
Valerian	*Arcobacter butzleri*		
Apple	*Arcobacter* spp.	10/50	[47]
Lettuce	*Cronobacter* spp.	122/403	[49]
Coriander			
Tomato			
Cucumber			
Vegetables	*Cronobacter turicensis,*	71/602	[51]
	*Cronobacter muytjensii*		
Vegetable	*Cronobacter sakazaki*	-	[52]
Spinach	*Arcobacter butzleri*	119/175	[54]
Lettuce	*Arcobacter cryaerophilus*		
Rocket	*Arcobacter butzleri*		
Valerian	*Arcobacter butzleri*		

“Occurrence” refers to the frequency or prevalence of *Arcobacter* spp. or *Cronobacter* spp. in the different fresh vegetables.

**Table 2:** Application of organic acids and chloride as emerging chemical methods for the inactivation of *Arcobacter* and *Cronobacter* spp.

Microorganism	Organic Acid and Chlorine	Matrix	Condition	Microbial Reduction	Potential Research Limitation	Reference
*C. sakazakii*	Chlorine, chlorine dioxide, and a peroxyacetic acid-based sanitiser	Surface of apples, cantaloupes, strawberries, lettuce, and tomatoes	Chlorine dioxide, and a peroxyacetic acid-based sanitiser (Tsunami 200) treatments (1 and 5 min).	Chlorine and chlorine dioxide 3.38 and 3.77 Log_10_ CFU/apple. Tsunami 200: 4 Log_10_ CFU/apple. Reductions of > or = 3.70 Log CFU/tomato with 10 ugml chlorine or chlorine dioxide or 40 microg/mL Tsunami 200 for 5 min. Treatment of lettuce with Tsunami 200 (40 and 80 microg/mL) reduction of 5.31 Log_10_ CFU/sample.	Only two treatment times (1 and 5 min) were evaluated.	[25]
*Arcobacter butzleri* and *Arcobacter* *cryaerophilus*	Propionic acid, lactic acid, malic acid, ascorbic acid, formic acid, and tartaric acid.	In vitro test	Propionic, lactic, malic, and ascorbic acids (pH 5.5–5.0), formic, citric, and tartaric acids in the pH range of 6.0–5.5	*Arcobacter butzleri* grows at pH 5.5. No viable cells were detected in BHI broth acidified to pH 5.0. with ascorbic, malic, lactic, or propionic acid; no growth observed at pH < 5.5. *Arcobacter cryaerophilus*: more susceptible to the decrease in pH values, regardless of the acidulant used.	The effect of the pH of brain heart infusion (BHI) broth as a positive control.	[26]
*C. sakazakii*	Para-hydroxybenzoate (BPB) Malic acid	Apple Juice	pH values of 3.2 to 9.0, supplemented with selected concentrations of BPB ≤ 125 ppm and thermally treated (58 °C).	6-Log_10_ reduction at 600 s, 125 ppm of BPB resulted in a 6-Log_10_ reduction in 30 s.	The effect on food quality has not been evaluated.	[31]
*C. sakazakii*	Acetic acid Propionic acid Malic acid	In vitro test	Acidic acetic acid, propionic acid, malic acid, and heat stress (55 °C).	The order of resistance of the acid-shocked *C. sakazakii* to the organic acids was acetic acid > propionic acid > malic acid.	The effect of pH has not been evaluated.	[32]

**Table 4:** Emerging physical methods for the inactivation of *Arcobacter* spp. and *Cronobacter* spp.

Microorganism	Emerging Physical	Matrix	Condition	Microbial Reduction	Potential Research Limitation	Reference
*Cronobacter sakazakii*	Combined ultrasound with organic acids	In situ test	Single treatment of ultrasound (frequency of 37 kHz and a power up to 380 W) treated with ultrasound alone for 5, 20, 40, 60, 80, and 100 min) or NaOCl 12% diluted with tap water to a final volume 80 mL and final concentrations of 50, 100, 150, and 200 ppm for 5 min. Combined ultrasound and NaOCl treatment, 24 combination treatments were compared with ultrasound (5, 20, 40,60, 80, and 100 min) and NaOCl (50, 100, 150, and 200 ppm for 5 min).	Despite the significant reduction in *C. sakazakii* with NaOCl treatment (200 ppm), the combination of 100 min ultrasound and 200 ppm NaOCl resulted in an additional 1.67 log-reduction in *C. sakazakii* (4.44 Log_10_ reduction = 2.77 + 1.67 Log_10_).	The minimum inhibitory concentration (MIC) and minimum bactericidal concentration (MBC) were not determined.	[34]
*Cronobacter sakazakii*	Ultraviolet-C (UV-C) radiation, acidic electrolysed (AEW), and neutral electrolysed (NEW) waters	In situ test	The fruits were contaminated by dip inoculation, contaminated mangoes were disinfected using UV-C (2.5, 5, 7.5, and 10 kJ/m^2^), AEW, NEW, and sodium hypochlorite (SH), and the microorganisms were monitored.	*C. sakazakii* grew after and UV-C was more effective in reducing *C. sakazakii* (2.6 Log CFU/g) when compared with AEW, NEW, and SH (1.8 Log CFU/g).	The combined effect of AEW, NEW, and UV-C was not evaluated.	[37]

## Data Availability

No new data were created or analyzed in this study.
